# Mental Treatment

**Published:** 1923-04

**Authors:** 


### MENTAL TREAMENT.

### THE GOVERNMENT AND AFTER CARE.

THE annual meeting of the Mental After-Care Association for poor persons convalescent or recovered from institutions for the insane took place at the Merchant Taylors' Hall, Threadneedle Street, on March 16, the chair being taken by Colonel Sir Charles Wakefield, Bt., the President of the Association. In his opening remarks the Chairman reminded the meeting how many poor people, on being discharged from mental hospitals, found the world strange and unfriendly, and needed sympathy and assistance to recover their mental poise and normal health. The Association had been formed for this purpose, and this year there was again a marked increase in the number of cases assisted.

### What the Government are Doing.

The Lord Chancellor, Viscount Cave, in moving the adoption of the report, said how glad he was to have an opportunity of expressing his sympathy with the work of the Association. The Government was giving special attention to the care of mentally afflicted persons, and he had reason to believe that proposals would shortly be made for providing better machinery for the treatment of cases of doubtful character. He strongly felt, too, that more attention ought to be paid to the after-care of those recovered, and this point also was receiving Government attention. Few positions were more pathetic than that of mental patients discharged without friends or anyone to give them a helping hand. Often they lacked clothes and food, and more especially did they need advice and help in obtaining employment.

### Work of the Association.

In seconding the motion, Sir Frederick Willis, Chairman of the Board of Control, emphasised the great value of the work of the Association, and expressed a desire to see it largely extended. About 10,000 poor people were discharged annually from mental hospitals, and of these the Association got into touch with about one thousand. Something should be done for the remainder, and he trusted that the Visiting Committees of the County Mental Hospitals would be given power to act in conjunction with the Association, which was so eminently fitted for the work. With regard to the accounts, Sir Maurice Craig, the Treasurer, said that they had for the first time received help from King Edward's Hospital Fund, as well as further assistance from Queen Adelaide Fund, the Queen Alexandra Day Fund, and the Guild of Help, while the Board of Control and the public authorities had assisted.

### The "Eternal Want of Pence."

Sir James Crichton-Browne emphasised the need for supporting and fortifying the Association, which supplied succour to the helpless, and whose reports showed a record of strenuous endeavour and increasing usefulness. The Association was hampered by want of money, and would have to curtail its operations unless liberally aided.

